# Supplementary figures and images for: Testing models of reciprocal relations between social influence and integration in STEM across the college years
Source: PLoS One. 2020 Sep 16;15(9):e0238250. doi: 10.1371/journal.pone.0238250 (PMC7494109; doi:10.1371/journal.pone.0238250)

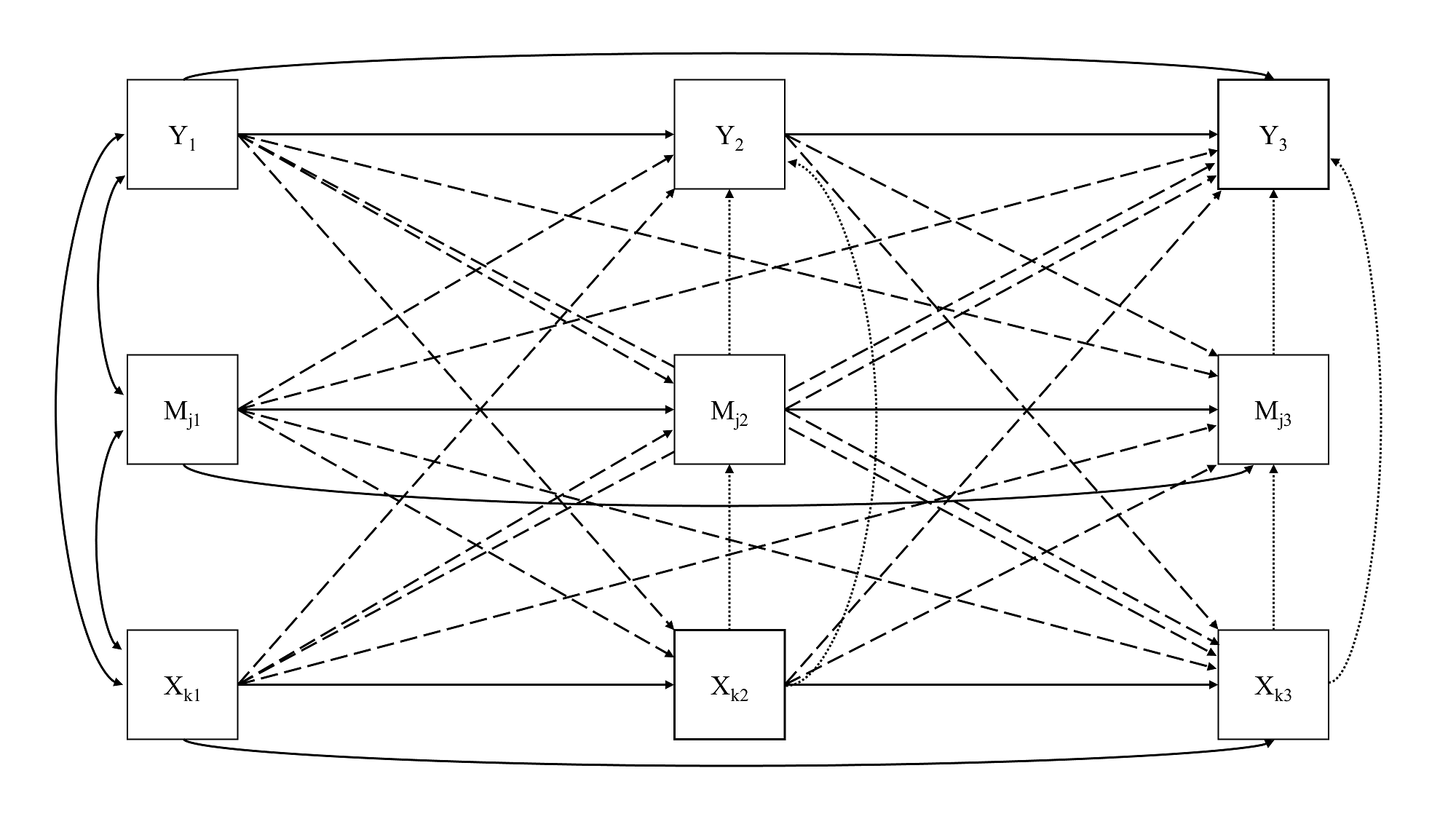

Supplement: S1 Fig — Y = outcome (i.e., scientific career persistence intentions [indicator of integration]), Mj = mediators (i.e., science self-efficacy, science identity, and science community values [indicators of social influence processes]), Xk = contextual factors (i.e., faculty mentor support, mentor network diversity, research experiences [indicators of social influence agents]). Stability paths shown as solid lines, first- and higher-order longitudinal cross-lagged paths shown as dashed lines, and contemporaneous mediation paths from the contextual factors to the outcome are shown as dotted lines. Subscripts 1 = T1 spring/summer prior to college 2015, 2 = T2 spring first year of college 2016, 3 = T3 spring second year of college 2017, but T4 fall third year of college (2017) and T5 spring fourth year of college (2018) are not shown for the sake of parsimony. (TIF) [file pone.0238250.s001.tif]

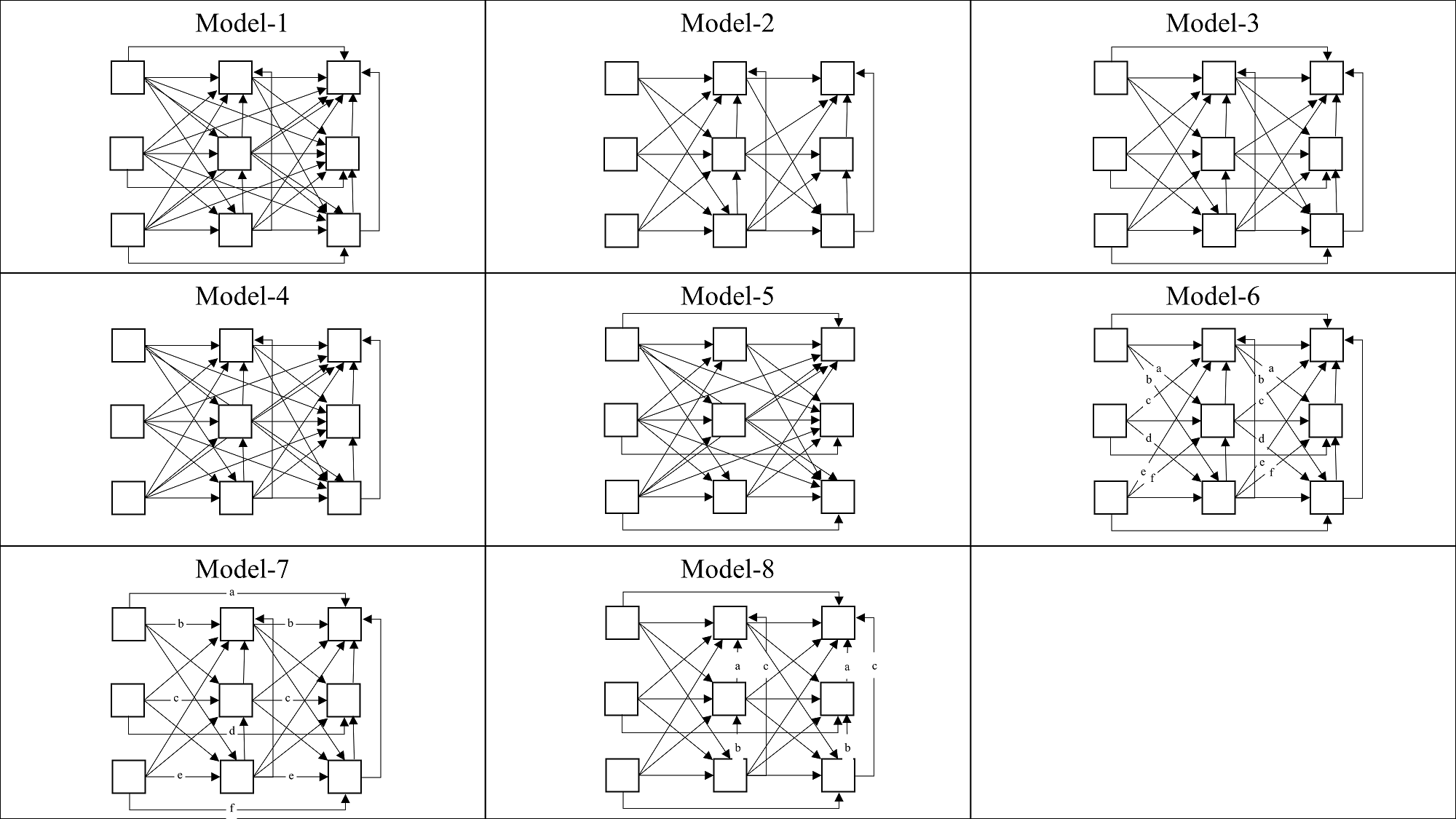

Supplement: S2 Fig — Model-1 represents the full-forward (i.e., first- and higher-order stability and cross-lagged paths) plus contemporaneous mediation model. Model-2 represents only first-order stability and cross-lagged plus contemporaneous mediation model. Model-3 represents first- and higher-order stability and first-order cross-lagged plus contemporaneous mediation model. Model-4 represents first- and higher-order cross-lagged and first-order stability plus contemporaneous mediation model. Model-5 represents the full forward without contemporaneous mediation model. Model-6 represents developmental equilibrium of the cross-lagged coefficients (i.e., invariance constraints) placed on the simplest and best fitting model identified from Models 1–5. Model-7 represents developmental equilibrium of the stability coefficients added to the simplest and best fitting model from Models 1–6. Model-8 represents contemporaneous equilibrium of the contemporaneous/cross-sectional coefficients added to the simplest and best fitting model from Models 1–7. Correlations among variables not shown for the sake of parsimony. (TIF) [file pone.0238250.s002.tif]

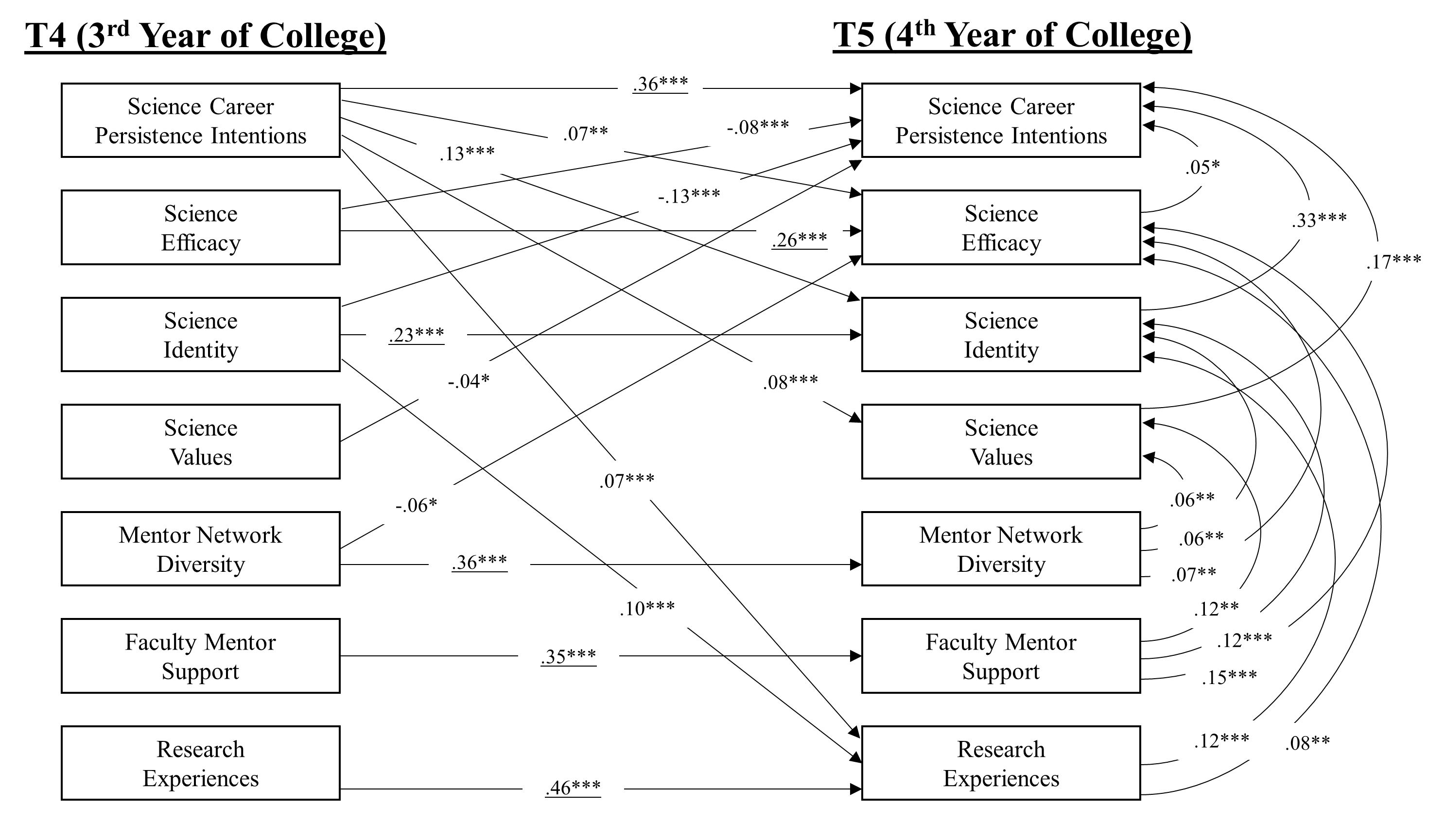

Supplement: S3 Fig — Only statistically significant paths are shown from coefficients in Table 2. All standardized structural coefficients ascertained from STDXY in Mplus as all variables were continuous. Underlined values represent stability coefficients; coefficients in standard text associated with predictors from the prior year in college are first-order cross-lagged coefficients, and coefficients associated with predictors from the current year of college are contemporaneous. The B-H FDR procedure was used to determine the statistical significance of all unstandardized coefficients. Based on the FDR procedure, all p-values less than .023 are reported statistically significant. *p≤.023, **p≤.01, ***p≤.001. (TIF) [file pone.0238250.s003.tif]
