# Supplementary material for: Testing models of reciprocal relations between social influence and integration in STEM across the college years
Source: PLoS One. 2020 Sep 16;15(9):e0238250. doi: 10.1371/journal.pone.0238250 (PMC7494109; doi:10.1371/journal.pone.0238250)
Supplement: S1 Table — Cronbach’s alphas are presented on the diagonals. *p ≤ .05, **p ≤ .01, ***p ≤ .001. (PDF) [file pone.0238250.s004.pdf]

S1 Table. Summary of descriptive statistics and correlations between predictors, mediators, and outcomes across time.

Variable	1.	2.	3.	4.	5.	6.	7.	8.	9.	10.	11.	12.
1. Female	--											
2. Science Efficacy (T1)	-.12**	*.81*										
3. Science Efficacy (T2)	-.12**	.40***	*.90*									
4. Science Efficacy (T3)	-.15***	.37***	.55***	*.89*								
5. Science Efficacy (T4)	-.05	.38***	.44***	.50***	*.88*							
6. Science Efficacy (T5)	-.03	.36***	.33***	.47***	.19***	*.89*						
7. Science Identity (T1)	-.07	.48***	.32***	.34***	.22***	.24***	*.88*					
8. Science Identity (T2)	-.09	.24***	.50***	.38***	.29***	.31***	.56***	*.88*				
9. Science Identity (T3)	-.07	.26***	.33***	.53***	.37***	.34***	.51***	.58***	*.90*			
10. Science Identity (T4)	-.03	.24***	.32***	.36***	.52***	.43***	.38***	.51***	.61***	*.90*		
11. Science Identity (T5)	.03	.24***	.29***	.27***	.36***	..55***	.38***	.53***	.51***	.58***	*.91*	
12. Science Community Values (T1)	-.11**	.45***	.30***	.29***	.15**	.14**	.64***	.40***	.39***	.29***	.23***	*.88*
13. Science Community Values (T2)	-.08	.24***	.49***	.33***	.29***	.22***	.44***	.60***	.45***	.35***	.40***	.49***
14. Science Community Values (T3)	-.08	.24***	.32***	.44***	.33***	.28***	.39***	.43***	.61***	.46***	.36***	.49***
15. Science Community Values (T4)	-.05	.15**	.25***	.24***	.42***	.28***	.28***	.32***	.40***	.58***	.42***	.32***
16. Science Community Values (T5)	-.01	.20***	.30***	.23***	.22***	.38***	.19***	.31***	.32***	.31***	.49***	.23***
17. Scientific Career Intentions (T1)	.07	.33***	.16***	.23***	.12**	.17***	.68***	.43***	.41***	.30***	.30***	.51***
18. Scientific Career Intentions (T2)	.04	.15**	.32***	.20***	.21***	.22***	.41***	.56***	.42***	.40***	.39***	.28***
19. Scientific Career Intentions (T3)	.07	.15**	.18***	.24***	.21***	.22***	.35***	.33***	.54***	.41***	.36***	.30***
20. Scientific Career Intentions (T4)	.08	.14**	.21***	.16***	.35***	.27***	.36***	.37***	.43***	.61***	.41***	.22***
21. Scientific Career Intentions (T5)	.13**	.16**	.16**	.19***	.25***	.40***	.23***	.27***	.39***	.44***	.60***	.14***
22. Mentor Network Diversity (T1)	.15***	.05	.05	.05	-.04	.03	.10*	.16**	.03	.02	.06	.09**
23. Mentor Network Diversity (T2)	.06	.01	.15***	.07	.05	.08	.09	.17***	.09	.07	.12*	.02
24. Mentor Network Diversity (T3)	.12**	.07	.00	.13**	.06	.08	.06	.08	.16***	.14**	.15**	.03
25. Mentor Network Diversity (T4)	.14***	.05	.03	.11*	.07	.06	.10*	.12*	.19***	.17***	.10*	.07
26. Mentor Network Diversity (T5)	.17***	.02	.01	.03	.08	.16***	.05	.06	.14**	.18***	.22***	-.01
27. Faculty Mentoring Practices (T2)	-.09	.09	.33***	.21*	.14	.03	.08	.30***	.15	-.02	.11	.08
28. Faculty Mentoring Practices (T3)	-.02	.14	.21*	.23***	.12	.22**	.16*	.10	.10	.12	.17*	.15*
29. Faculty Mentoring Practices (T4)	.05	.10	.13	.20**	.24***	.23**	.07	.04	.18*	.20**	.15*	.09
30. Faculty Mentoring Practices (T5)	.02	.03	.14	.15*	.01	11	.01	-.06	.00	.00	.03	.01
31. Research Experiences (T2)	.10*	.08	.14	.10*	.04	.07	.20***	.22***	.07	.07	.08	.12*
32. Research Experiences (T3)	.08	.09	.10*	.18***	.11*	.13**	.20***	.23***	.24***	.18***	.21***	.17***
33. Research Experiences (T4)	.05	.19***	.06	.17***	.16***	.19***	.25***	.25***	.28***	.29***	.29***	.20***
34. Research Experiences (T5)	.06	.15**	.04	.11*	.09*	.20***	.20***	.20***	.31***	.32***	.35***	.16***

Table continues…

S1 Table continued…

Variable	13.	14.	15.	16.	17.	18.	19.	20.	21.	22.	23.	24.
13. Science Community Values (T2)	*.91*											
14. Science Community Values (T3)	.54***	*.91*										
15. Science Community Values (T4)	.47***	.53***	*.89*									
16. Science Community Values (T5)	.38***	.37***	.37***	*.87*								
17. Scientific Career Intentions (T1)	.32***	.32***	.19***	.12*	*.71*							
18. Scientific Career Intentions (T2)	.55***	.40***	.35***	.27***	.51***	*.73*						
19. Scientific Career Intentions (T3)	.36***	.52***	.32***	.24***	.44***	.57***	*.70*					
20. Scientific Career Intentions (T4)	.34***	.40***	.49***	.24***	.43***	.54***	.61***	*.68*				
21. Scientific Career Intentions (T5)	.30***	.34***	.37***	.33***	.34***	.46***	.56***	.59***	*.61*			
22. Mentor Network Diversity (T1)	.04	.08	-.06	-.01	.06	.16**	.07	.07	.11*	--		
23. Mentor Network Diversity (T2)	.14**	.04	.02	.01	.08	.15**	.08	.08	.04	.27***	--	
24. Mentor Network Diversity (T3)	.05	.13**	.06	.10*	.11*	.10*	.12**	.13**	.14**	.24***	.42***	--
25. Mentor Network Diversity (T4)	.09	.16***	.15***	.13**	.09*	.11*	.15***	.15***	.18***	.01	.32***	.51***
26. Mentor Network Diversity (T5)	.02	.09	.14**	.09	.04	.13*	.13**	.18***	.25***	.28***	.30***	.39***
27. Faculty Mentoring Practices (T2)	.22*	.12	-.07	.20	.06	.13	.03	-.02	.13	.01	.09	-.01
28. Faculty Mentoring Practices (T3)	.12	.11	.07	.09	.08	.06	.08	.08	.14	.05	.01	.15*
29. Faculty Mentoring Practices (T4)	.07	.27***	.29***	.10	.00	.04	.23***	.19**	.26**	.05	.08	.30***
30. Faculty Mentoring Practices (T5)	-.02	.00	.06	.07	.00	.12	-.03	.03	07	.09	.10	.20**
31. Research Experiences (T2)	.17	.16***	.06	.07	.19***	.18***	.13*	.13**	.15**	.16**	.23***	.24***
32. Research Experiences (T3)	.18***	.22***	.11*	.17***	.21***	.20***	.20***	.19***	.18***	.09	.19***	.31***
33. Research Experiences (T4)	.22***	.24***	.19***	.16***	.23***	.23***	.29***	.28***	.29***	.13***	.21***	.33***
34. Research Experiences (T5)	.19***	.22***	.22***	.21***	.21***	.23***	.30***	.26***	.40***	.14**	.13**	.28***

Table continues…

S1 Table continued…

Variable	25.	26.	27.	28.	29.	30.	31.	32.	33.	34.
25. Mentor Network Diversity (T4)	--									
26. Mentor Network Diversity (T5)	.51***	--								
27. Faculty Mentoring Practices (T2)	.03	.02	*.92*							
28. Faculty Mentoring Practices (T3)	.15*	.19*	.25*	*.88*						
29. Faculty Mentoring Practices (T4)	.24***	.23***	.08	.49***	*.90*					
30. Faculty Mentoring Practices (T5)	.22***	.17**	.05	.28***	.41***	*.91*				
31. Research Experiences (T2)	.20***	.13*	.12	.04	.07	.12	--			
32. Research Experiences (T3)	.30***	.15**	.10	.03	.00	.02	.39***	--		
33. Research Experiences (T4)	.40***	.26***	.11	.05	.11	.00	.32***	.67***	--	
34. Research Experiences (T5)	.36***	.36***	-.03	.03	.17*	.11	.22***	.46***	.59***	--

S1 Table Notes: Cronbach's alphas are presented on the diagonals. $*p \leq .05$, $**p \leq .01$, $***p \leq .001$
