# Supplementary material for: Testing models of reciprocal relations between social influence and integration in STEM across the college years
Source: PLoS One. 2020 Sep 16;15(9):e0238250. doi: 10.1371/journal.pone.0238250 (PMC7494109; doi:10.1371/journal.pone.0238250)
Supplement: S2 Table — T1 = pre-college, T2 = spring 1st year of college, T3 = spring 2nd year of college, T4 = fall 3rd year of college, T5 = spring 4th year of college. aThe residual variance of item-2 at time-1 of the persistence intentions scale was not different from zero which causes convergence problems. Therefore, the variance of item-2 at time-1 was constrained to zero as were all residual correlations with the item. bThe residual variance of item-2 at times 1, 2, & 3 of the persistence intentions scale was not different from zero which causes convergence problems. Therefore, the variance of item-2 at times 1–3 was constrained to zero as were all residual correlations with the item. *p ≤ .05, **p ≤ .01, ***p ≤ .001. (PDF) [file pone.0238250.s005.pdf]

**S2 Table. Summary of longitudinal confirmatory factor analyses (N=751).**

| *Model* | $\chi^2(df)$ | *CFI* | *RMSEA 90% CI* | *Model Comparison* | *Pass?* |
|---|---|---|---|---|---|
| *Longitudinal Measurement Invariance* | | | | | |
| *Science Efficacy* | | | | | |
| Men | | | | | |
| 1.11 Configural Invariance | 470.25 (335) | .957 | .036 [.028, .043] | | |
| 1.12 Metric Invariance | 492.17 (355) | .956 | .035 [.027, .042] | 1.11 vs 1.12 | Y |
| Women | | | | | |
| 1.21 Configural Invariance | 499.44 (335) | .958 | .034 [.027, .040] | | |
| 1.22 Metric Invariance | 513.56 (355) | .960 | .032 [.026, .038] | 1.21 vs 1.22 | Y |
| *Science Identity* | | | | | |
| Men | | | | | |
| 2.11 Configural Invariance | 323.45 (215) | .968 | .040 [.031, .048] | | |
| 2.12 Metric Invariance | 347.91 (231) | .966 | .040 [.021, .048] | 2.11 vs 2.12 | Y |
| Women | | | | | |
| 2.21 Configural Invariance | 293.75 (215) | .981 | .029 [.020, .037] | | |
| 2.22 Metric Invariance | 316.93 (231) | .979 | .029 [.021, .037] | 2.21 vs 2.22 | Y |
| *Science Community Values* | | | | | |
| Men | | | | | |
| 3.11 Configural Invariance | 146.89 (120)* | .988 | .027 [.003, .040] | | |
| 3.12 Metric Invariance | 170.09 (132)* | .983 | .030 [.014, .042] | 3.11 vs 3.12 | Y |
| Women | | | | | |
| 3.21 Configural Invariance | 140.10 (120) | .993 | .020 [.000, .032] | | |
| 3.22 Metric Invariance | 161.41 (132)* | .989 | .023 [.005, .034] | 3.21 vs 3.22 | Y |

Table continues…

S2 Table continued…

| *Model* | $\chi^2$*(df)* | *CFI* | *RMSEA 90% CI* | *Model Comparison* | *Pass?* |
|---|---|---|---|---|---|
| *Persistence Intentions* | | | | | |
| Men | | | | | |
| 4.11 Configural Invariance | 181.83 (120)*** | .960 | .040 [.028, .052] | | |
| 4.12 Metric Invariance | 231.84 (132)*** | .935 | .049 [.038, .059] | 4.11 vs 4.12 | N |
| 4.13 Partial Metric Invariance | 188.31 (129)*** | .961 | .038 [.025, .049] | 4.11 vs 4.13 | Y |
| Women | | | | | |
| 4.21 Configural Invariance | 182.96 (120)*** | .969 | .035 [.024, .045] | | |
| 4.22 Metric Invariance | 270.15 (132)*** | .933 | .049 [.041, .058] | 4.21 vs 4.22 | N |
| 4.23 Partial Metric Invariance | 202.24 (129)*** | .964 | .036 [.026, .046] | 4.21 vs 4.23 | Y |
| *Cross-Gender Measurement Invariance* | | | | | |
| *T1* | | | | | |
| 5.1 Configural Invariance | 509.48 (292)*** | .954 | .049 [.042, .056] | | |
| 5.2 Metric Invariance | 523.43 (307)*** | .954 | .048 [.041, .055] | 5.1 vs 5.2 | Y |
| 5.3 Correlation Invariance | 528.65 (313)*** | .954 | .047 [.040, .054] | 5.1 vs 5.3 | Y |
| *T2* | | | | | |
| 6.1 Configural Invariance | 596.96 (292)*** | .926 | .067 [.059, .075] | | |
| 6.2 Metric Invariance | 629.67 (307)*** | .921 | .067 [.060, .075] | 6.1 vs 6.2 | Y |
| 6.3 Correlation Invariance | 629.21 (313)*** | .923 | .066 [.059, .074] | 6.1 vs 6.3 | Y |
| *T3* | | | | | |
| 7.1 Configural Invariance | 498.05 (292)*** | .955 | .051 [.043, .058] | | |
| 7.2 Metric Invariance | 508.12 (307)*** | .956 | .049 [.041, .056] | 7.1 vs 7.2 | Y |
| 7.3 Correlation Invariance | 515.36 (313)*** | .955 | .049 [.041, .056] | 7.1 vs 7.3 | Y |
| *T4* | | | | | |
| 8.1 Configural Invariance | 539.75 (292)*** | .945 | .056 [.048, .063] | | |
| 8.2 Metric Invariance | 562.02 (307)*** | .944 | .055 [.048, .062] | 8.1 vs 8.2 | Y |
| 8.3 Correlation Invariance | 562.90 (313)*** | .945 | .054 [.047, .061] | 8.1 vs 8.3 | Y |

S2 Table continued…

| *Model* | $\chi^2$*(df)* | *CFI* | *RMSEA 90% CI* | *Model Comparison* | *Pass?* |
|---|---|---|---|---|---|
| *T5* | | | | | |
| 9.1 Configural Invariance | 496.94 (292)*** | .940 | .055 [.047, .063] | | |
| 9.2 Metric Invariance | 514.85 (307)*** | .940 | .054 [.046, .062] | 9.1 vs 9.2 | Y |
| 9.3 Correlation Invariance | 519.00 (313)*** | .940 | .053 [.045, .061] | 9.1 vs 9.3 | Y |

S2 Table Notes: T1 = pre-college, T2 = spring 1st year of college, T3 = spring 2nd year of college, T4 = fall 3rd year of college, T5 = spring 4th year of college.

*$p \leq .05$, **$p \leq .01$, ***$p \leq .001$
