# Supplementary material for: Testing models of reciprocal relations between social influence and integration in STEM across the college years
Source: PLoS One. 2020 Sep 16;15(9):e0238250. doi: 10.1371/journal.pone.0238250 (PMC7494109; doi:10.1371/journal.pone.0238250)
Supplement: S3 Table — Model 1 T-size results for equivalent testing were as follows (a) CFI = .970 and (b) RMSEA = .045; Model 1 T-size results relative to descriptive cutoff values were as follows for CFI (a) Excellent = .989, (b) Close = .950, (c) Fair = .920 and for RMSEA (a) Excellent = .022, (b) Close = .057, (c) Fair = .087; Model 8 T-Size results for equivalent testing were as follows (a) CFI = .946 and (b) RMSEA = .032; Model 8 T-size results relative to descriptive cutoff values were as follows for CFI (a) Excellent = .985, (b) Close = .942, (c) Fair = .909 and for RMSEA (a) Excellent = .017, (b) Close = .055, (c) Fair = .088. ***p ≤ .001. (PDF) [file pone.0238250.s006.pdf]

S3 Table. Summary of model fit and model comparison statistics for nested longitudinal mediation analysis models (N=751).

Model	χ^2 *(df)*	*CFI*	*SRMR*	*RMSEA [90% CI]*	*Model Comparison (Pass?)*
1. Full forward cross-lagged panel model + contemporaneous mediation paths; all paths freely estimated.	212.67 (104)***	.982	.043	.037 [.030, .044]	n/a
2. Only first-order stability and cross-lagged panel + contemporaneous mediation; all paths freely estimated.	827.29 (296)***	.914	.084	.049 [.045, .053]	1 vs 2 (No)
3. First- and higher-order stability, but first-order cross-lagged + contemporaneous mediation; all paths freely estimated.	418.88 (260)***	.974	.052	.029 [.023, .033]	1 vs 3 (Yes)
4. First-order stability, but first- and higher-order cross-lagged + contemporaneous mediation; all paths freely estimated.	563.51 (140)***	.931	.056	.063 [.058, .069]	1 vs 4 (No)
5. Full forward cross-lagged panel model + no contemporaneous mediation; all paths freely estimated.	698.22 (158)***	.912	.074	.067 [.062, .073]	1 vs 5 (No)
6. First- and higher-order stability, but first-order cross-lagged + contemporaneous mediation; invariance of cross-lagged paths across time (developmental equilibrium).	521.75 (342)***	.971	.056	.026 [.022, .031]	3 vs 6 (Yes)
7. First- and higher-order stability, but first-order cross-lagged + contemporaneous mediation; invariance of cross-lagged and stability paths across time (developmental + stability equilibrium).	675.61 (378)***	.952	.068	.032 [.028, .036]	6 vs 7 (No)
8. First- and higher-order stability, but first-order cross-lagged + contemporaneous mediation; invariance of cross-lagged and contemporaneous paths across time (developmental + contemporaneous equilibrium).	608.65 (387)***	.964	.060	.028 [.023, .032]	6 vs 8 (Yes)

S3 Table Notes: Model 1 T-size results for equivalent testing were as follows (*a*) CFI = .970 and (*b*) RMSEA = .045; Model 1 T-size results relative to descriptive cutoff values were as follows for CFI (*a*) Excellent = .989, (*b*) Close = .950, (*c*) Fair = .920 and for RMSEA (*a*) Excellent = .022, (*b*) Close = .057, (*c*) Fair = .087; Model 8 T-Size results for equivalent testing were as follows (*a*) CFI = .946 and (*b*) RMSEA = .032; Model 8 T-size results relative to descriptive cutoff values were as follows for CFI (*a*) Excellent = .985, (*b*) Close = .942, (*c*) Fair = .909 and for RMSEA (*a*) Excellent = .017, (*b*) Close = .055, (*c*) Fair = .088.

***$p \leq .001$
