# Supplementary material for: Testing models of reciprocal relations between social influence and integration in STEM across the college years
Source: PLoS One. 2020 Sep 16;15(9):e0238250. doi: 10.1371/journal.pone.0238250 (PMC7494109; doi:10.1371/journal.pone.0238250)
Supplement: S4 Table — T1 = pre-college, T2 = spring 1st year of college, T3 = spring 2nd year of college, T4 = fall 3rd year of college, T5 = spring 4th year of college. All standardized structural coefficients ascertained from STDXY in Mplus as all variables were continuous. Underlined values represent stability coefficients, coefficients in standard text associated with predictors from the pre-college are first-order cross-lagged coefficients, and coefficients associated with predictors from the 1st year of college are contemporaneous. The B-H FDR procedure was used to determine the statistical significance of all unstandardized coefficients. Based on the FDR procedure, all p-values less than .023 for unstandardized coefficients are reported statistically significant. *p≤.023, **p≤.01, ***p≤.001. (PDF) [file pone.0238250.s007.pdf]

S4 Table. Summary of standardized structural coefficients predicting engagement with social influence agents, social influence processes, and integration into STEM in the first-year of college (Model 8, N=751).

		T2 1st Year of College Outcomes						
Time	*Predictors*	*Persistence Intentions*	*Science Efficacy*	*Science Identity*	*Science Values*	*Mentor Network Diversity*	*Faculty Mentor Support*	*Research Experiences*
T1 Pre-college	Persistence Intentions	.47***	.06**	.13***	.07***	-.03	-.02	.11***
	Science Efficacy	-.08***	.34***			-.02	.05	-.04
	Science Identity	-.14***		.42***		.06	-.05	.16***
	Science Values	-.06*			.43***	-.01	.06	.02
	Mentor Network Diversity	.02	-.07*	-.04	-.04	.24***		
T2 1st year of college (Contemporaneous)								
	Science Efficacy	.05*						
	Science Identity	.32***						
	Science Values	.21***						
	Mentor Network Diversity	.01	.06**	.06**	.05**			
	Faculty Mentor Support	-.01	.15***	.14***	.11**			
	Research Experiences	.03	.05**	.08***	.03			
R^2		.47	.18	.32	.25	.07	.01	.06

S4 Table Note: T1 = pre-college, T2 = spring 1st year of college, T3 = spring 2nd year of college, T4 = fall 3rd year of college, T5 = spring 4th year of college. All standardized structural coefficients ascertained from STDXY in Mplus as all variables were continuous. Underlined values represent stability coefficients, coefficients in standard text associated with predictors from the pre-college are first-order cross-lagged coefficients, and coefficients associated with predictors from the 1st year of college are contemporaneous. The B-H FDR procedure was used to determine the statistical significance of all unstandardized coefficients. Based on the FDR procedure, all *p*-values less than .023 for unstandardized coefficients are reported statistically significant.

*$p \leq .023$, **$p \leq .01$, ***$p \leq .001$
