# Supplementary material for: Testing models of reciprocal relations between social influence and integration in STEM across the college years
Source: PLoS One. 2020 Sep 16;15(9):e0238250. doi: 10.1371/journal.pone.0238250 (PMC7494109; doi:10.1371/journal.pone.0238250)
Supplement: S5 Table — All standardized structural coefficients ascertained from STDXY in Mplus as all variables were continuous. Underlined values represent stability coefficients, coefficients in standard text associated with predictors from the pre-college are first-order cross-lagged coefficients, and coefficients associated with predictors from the 1st year of college are contemporaneous. The B-H FDR procedure was used to determine the statistical significance of all unstandardized coefficients. Based on the FDR procedure, all p-values less than .023 for unstandardized coefficients are reported statistically significant. *p≤.023, **p≤.01, ***p≤.001. (PDF) [file pone.0238250.s008.pdf]

S5 Table. Summary of standardized structural coefficients for social influence factors, social influence processes, and integration in the second-year of college (Model 8, N=751).

		T3 2nd Year of College Outcomes						
Time	*Predictors*	*Persistence Intentions*	*Science Efficacy*	*Science Identity*	*Science Values*	*Mentor Network Diversity*	*Faculty Mentor Support*	*Research Experiences*
T1 or Pre-college	Persistence Intentions	.08						
	Science Efficacy		.19***					
	Science Identity			.22***				
	Science Values				.28***			
	Mentor Network Diversity					.16***		
T2 or 1st year of college	Persistence Intentions	.42***	.07**	.14***	.08***	.03	-.02	.10***
	Science Efficacy	-.09***	.42***			-.02	.07	-.04
	Science Identity	-.13***		.36***		.05	-.05	.12***
	Science Values	-.06*			.36***	-.01	.07	.02
	Mentor Network Diversity	.02	-.06*	-.03	-.03	.35***		
	Faculty Mentor Support	.05	-.03	-.04	-.05		.25*	
	Research Experiences	.003	-.01	-.04	-.02			.30***
T3 or 2nd year of college (Contemporaneous)	Science Efficacy	.05*						
	Science Identity	.32***						
	Science Values	.19***						
	Mentor Network Diversity	.01	.07**	.06**	.05**			
	Faculty Mentor Support	-.01	.15***	.13***	.11**			
	Research Experiences	.04	.06**	.10***	.04			
R^2		.49	.36	.41	.38	.18	.08	.15

S5 Table Note: All standardized structural coefficients ascertained from STDXY in Mplus as all variables were continuous. Underlined values represent stability coefficients, coefficients in standard text associated with predictors from the pre-college are first-order cross-lagged coefficients, and coefficients associated with predictors from the 1st year of college are contemporaneous. The B-H FDR procedure was used to determine the statistical significance of all unstandardized coefficients. Based on the FDR procedure, all *p*-values less than .023 for unstandardized coefficients are reported statistically significant.

*$p \leq .023$, **$p \leq .01$, ***$p \leq .001$
