# Supplementary material for: Testing models of reciprocal relations between social influence and integration in STEM across the college years
Source: PLoS One. 2020 Sep 16;15(9):e0238250. doi: 10.1371/journal.pone.0238250 (PMC7494109; doi:10.1371/journal.pone.0238250)
Supplement: S6 Table — All standardized structural coefficients ascertained from STDXY in Mplus as all variables were continuous. Underlined values represent stability coefficients, coefficients in standard text associated with predictors from the pre-college are first-order cross-lagged coefficients, and coefficients associated with predictors from the 1st year of college are contemporaneous. The B-H FDR procedure was used to determine the statistical significance of all unstandardized coefficients. Based on the FDR procedure, all p-values less than .023 for unstandardized coefficients are reported statistically significant. *p≤.023, **p≤.01, ***p≤.001. (PDF) [file pone.0238250.s009.pdf]

S6 Table. Summary of standardized structural coefficients for social influence factors, social influence processes, and integration in the third-year of college (Model 8, N=751).

		T4 3rd Year of College Outcomes						
Time	*Predictors*	*Persistence Intentions*	*Science Efficacy*	*Science Identity*	*Science Values*	*Mentor Network Diversity*	*Faculty Mentor Support*	*Research Experiences*
T1 or Pre-college	Persistence Intentions	.13**						
	Science Efficacy		.17***					
	Science Identity			.01				
	Science Values				.04			
	Mentor Network Diversity					.08		
T2 1st year of college	Persistence Intentions	.15***						
	Science Efficacy		.19***					
	Science Identity			.29***				
	Science Values				.27***			
	Mentor Network Diversity					.10*		
	Faculty Mentor Support						-.05	
	Research Experiences							.07
T3 2nd year of college	Persistence Intentions	.38***	.07***	.14***	.11***	.03	-.02	.08***
	Science Efficacy	-.08***	.27***			-.02	.06	-.03
	Science Identity	-.13***		.30***		.05	-.05	.11***
	Science Values	-.06*			.27***	-.01	.06	.02
	Mentor Network Diversity	.02	-.06*	-.03	-.05	.41***		
	Faculty Mentor Support	.05	-.03	-.03	-.07		.55***	
	Research Experiences	.003	-.02	-.05	-.03			.58***
T4 3rd year of college (Contemporaneous)	Science Efficacy	.05*						
	Science Identity	.34***						
	Science Values	.13***						
	Mentor Network Diversity	.01	.07**	.06**	.08**			
	Faculty Mentor Support	-.01	.15***	.12***	.15**			
	Research Experiences	.05	.08**	.11***	.07			
R^2		.59	.33	.44	.35	.25	.32	.46

S6 Table Note: All standardized structural coefficients ascertained from STDXY in Mplus as all variables were continuous. Underlined values represent stability coefficients, coefficients in standard text associated with predictors from the pre-college are first-order cross-lagged coefficients, and coefficients associated with predictors from the 1st year of college are contemporaneous. The B-H FDR procedure was used to determine the statistical significance of all unstandardized coefficients. Based on the FDR procedure, all *p*-values less than .023 for unstandardized coefficients are reported statistically significant.

*$p \leq .023$, **$p \leq .01$, ***$p \leq .001$
