# Supplementary material for: Testing models of reciprocal relations between social influence and integration in STEM across the college years
Source: PLoS One. 2020 Sep 16;15(9):e0238250. doi: 10.1371/journal.pone.0238250 (PMC7494109; doi:10.1371/journal.pone.0238250)
Supplement: S7 Table — All standardized structural coefficients ascertained from STDXY in Mplus as all variables were continuous. Underlined values represent stability coefficients, coefficients in standard text associated with predictors from the pre-college are first-order cross-lagged coefficients, and coefficients associated with predictors from the 1st year of college are contemporaneous. The B-H FDR procedure was used to determine the statistical significance of all unstandardized coefficients. Based on the FDR procedure, all p-values less than .023 for unstandardized coefficients are reported statistically significant. *p≤.023, **p≤.01, ***p≤.001. (PDF) [file pone.0238250.s010.pdf]

S7 Table. Summary of standardized structural coefficients for social influence factors, social influence processes, and integration in the fourth-year of college (Model 8, N=751).

		T5 4th Year of College Outcomes						
Time	*Predictors*	*Persistence Intentions*	*Science Efficacy*	*Science Identity*	*Science Values*	*Mentor Network Diversity*	*Faculty Mentor Support*	*Research Experiences*
T1 Pre-college	Persistence Intentions	-.01						
	Science Efficacy		.11*					
	Science Identity			.04				
	Science Values				.03			
	Mentor Network Diversity					.12**		
T2 1st year of college	Persistence Intentions	.05						
	Science Efficacy		-.001					
	Science Identity			.23***				
	Science Values				.18**			
	Mentor Network Diversity					.11*		
	Faculty Mentor Support						.03	
	Research Experiences							-.02
T3 2nd year of college	Persistence Intentions	.20***						
	Science Efficacy		.26***					
	Science Identity			.14**				
	Science Values				.15**			
	Mentor Network Diversity					.10		
	Faculty Mentor Support						.12	
	Research Experiences							.13**
T4 3rd year of college	Persistence Intentions	.36***	.07**	.13***	.08***	.03	-.02	.07***
	Science Efficacy	-.08***	.26***			-.02	.06	-.03
	Science Identity	-.13***		.23***		.05	-.05	.10***
	Science Values	-.04*			.11	-.01	.04	.01
	Mentor Network Diversity	.02	-.06*	-.03	-.04	.36***		
	Faculty Mentor Support	.05	-.03	-.03	-.05		.35***	
	Research Experiences	.004	-.02	-.06	-.03			.46***
T5 4th year of college (Contemporaneous)	Science Efficacy	.05*						
	Science Identity	.33***						
	Science Values	.17***						
	Mentor Network Diversity	.01	.07**	.06**	.06**			
	Faculty Mentor Support	-.01	.15***	.12***	.12**			
	Research Experiences	.05	.08**	.12***	.06			

		T5 4th Year of College Outcomes						
Time	*Predictors*	*Persistence Intentions*	*Science Efficacy*	*Science Identity*	*Science Values*	*Mentor Network Diversity*	*Faculty Mentor Support*	*Research Experiences*
R^2		.58	.34	.43	.21	.28	.20	.37

S7 Table Note: All standardized structural coefficients ascertained from STDXY in Mplus as all variables were continuous. Underlined values represent stability coefficients, coefficients in standard text associated with predictors from the pre-college are first-order cross-lagged coefficients, and coefficients associated with predictors from the 1st year of college are contemporaneous. The B-H FDR procedure was used to determine the statistical significance of all unstandardized coefficients. Based on the FDR procedure, all *p*-values less than .023 for unstandardized coefficients are reported statistically significant.

**p*≤.023, ***p*≤.01, ****p*≤.001
