# Supplementary material for: Testing models of reciprocal relations between social influence and integration in STEM across the college years
Source: PLoS One. 2020 Sep 16;15(9):e0238250. doi: 10.1371/journal.pone.0238250 (PMC7494109; doi:10.1371/journal.pone.0238250)
Supplement: S1 File — (DOCX) [file pone.0238250.s011.docx]

**Supplemental Materials for Testing Models of Reciprocal Relations between Social Influence and Integration in STEM across the College Years**

The purpose of these supplemental materials is to provide additional background details that support the substantive analyses presented in the main narrative of this article. First we provide a comprehensive review of the logic and methodology for testing longitudinal and reciprocal mediation. Second, we provide additional details on the results. That is, we report on the results of

- the full correlation matrix including all observed scale-scored variables at all time points, as well as reliability estimates (S1 Table),
- the longitudinal confirmatory factor analyses (CFAs) of each construct to test measurement invariance over time – performed separately for men and women
  (S2 Table),
- the multiple group (i.e., men, women) CFAs for all constructs at each time point to test cross-group measurement invariance (S2 Table),
- the invariance of the correlations among constructs across groups (S2 Table)
- a summary of model fit and model comparison statistics for nested longitudinal models (S3 Table), and
- a summary of standardized structural coefficients predicting engagement with social influence agents, social influence processes, and integration into STEM across all time points (S4-S7 Tables).

**Longitudinal mediation framework**

As noted in the main text, we adopted a longitudinal analytic approach that would allow us to characterize both longitudinal and contemporaneous mediated effects. Longitudinal mediation is typically of most interest as it quantifies the long-term within-person changes in an outcome due to the indirect effect of a causal variable. In contrast, contemporaneous mediation quantifies the cross-sectional, between-person differences in an outcome due to the indirect effect of the causal variable (1). Simultaneously estimating both longitudinal and contemporaneous indirect effects takes advantage of time-ordering information to aid the determination of causal ordering and circumvents problems due to potential misalignment between the timing of measurement and the timing of the mediated effects (1). That is, longitudinal mediated effects are robustly estimated using research designs with three or more repeated measurement occasions in order to establish temporal precedence (i.e., the cause [X] is measured prior to the mediator [M], and M is measured prior to the outcome [Y]) (2, 3). However, longitudinal mediation may not detect a true mediated effect when the relationship among variables is more rapid than the time-lag between measurement occasions (1). For example, if impacts go up and down during the lag time between measurements, the analysis may show no or low effects, when effects were indeed occurring. Contemporaneous mediation, which focuses on cross-sectional effects, may more accurately reflect a true mediated effect when the timing of measurement and timing of mediation are misaligned (1). In the present study, we estimate both longitudinal and contemporaneous mediated effects as the timing of measurement is known (e.g., annually), but the timing of the mediated effect is unknown.

Consistent with best practices, we adopted a model comparison approach and conducted a series of eight nested structural equation models (SEMs) to systematically test for longitudinal, contemporaneous, and reciprocal mediation (1, 4-7). The first model (i.e., Model-1) is a “full forward” model in which constructs measured at earlier time points are predictors of constructs measured at all later time points (4), as well as contemporaneous mediated effects (i.e., MacKinnon’s auto-regressive mediation model III; MacKinnon, 2008). That is, Model-1 would provide optimal fit and parsimony if earlier engagement with social influence agents had immediate (contemporaneous), short-term (1-year later), and longer-term (2-4 years later) impacts on social influence processes and social integration and vice versa. S1 Figure presents a simplified three-wave representation of Model-1 (i.e., full forward model plus contemporaneous mediation). Model-1 tests the model-data fit of a SEM that

- predicts future status on each construct from prior status on the same construct (i.e., first- and higher-order auto-regression, hereafter referred to as “stability” paths; S1 Figure solid lines [e.g., Y_1_ → Y_2_ and Y_1_ → Y_3_]);
- predicts future status on each outcome, social influence process, and social influence agent from prior status on the other constructs (i.e., first- and higher-order cross-lagged paths; S1 Figure dashed lines [e.g., X_k1_ → M_j2_]);
- predicts contemporaneous mediation linking the social influence agents to social integration through the social influence processes (i.e., S1 Figure dotted lines [e.g., X_k2_ → M_j2_ → Y_2_]);
- estimates correlations among all variables at the first time point (i.e., S1 Figure solid double-headed lines [e.g., X_k1_ ↔ M_j1_]); and
- estimates correlations among social influence processes within each time point (e.g., Efficacy_Year2_ ↔ Identity_Year2_, not shown in S1 Figure for the sake of simplicity), as well as the correlations among the social influence agents within each time point (e.g., Mentor Network Diversity_Year2_ ↔ Research Experiences_Year2_, not shown in S1 Figure for the sake of simplicity).

The successive seven nested models add constraints to test the relative fit of more parsimonious longitudinal models (S1 Figure). Model-2 provides an extremely simplified alternative to Model-1, wherein there is no evidence of long-term (2-4 years later) stability or cross-lagged effects. Thus, Model-2 constrains all higher-order stability and cross-lagged paths to zero, but all other paths from Model-1 are freely estimated. S2 Figure depicts a simplified, three-wave version of Model-2. Models-3 and -4 test the fit of less extremely parsimonious alternatives. Specifically, Model-3 tests the fit of a model, wherein there is no evidence of long-term cross-lagged effects; that is all higher-order cross-lagged paths are constrained to zero (S2 Figure, Model-3). While Model-4 tests the fit of a model, wherein there is no evidence of long-term stability effects; that is all higher-order stability paths are constrained to zero (S2 Figure, Model-4). Model-5 tests the fit of a model wherein there is no evidence of contemporaneous mediated effects; that is all contemporaneous paths are constrained to zero (S2 Figure, Model-5).

The final three nested models test for simplifications based on developmental and contemporaneous equilibrium. Conceptually, equilibrium is defined as a state in which the relationships among a set of variables has reached temporal stability (8). Equilibrium is particularly important for mediation analysis, as temporal stability in the relationships among the predictor, mediator, and outcome (i.e., X, M, and Y) are essential for accurate estimates of indirect effects (1). In addition, testing and establishing the presence of equilibrium provides advantages related to protection against alternative interpretations due to unmeasured common causes of the variables in the analysis (9), consistency in the mutual influence among the constructs over time, parsimony of the model, precision of estimates, and ease of interpretation of the results (5). In longitudinal studies with three or more waves of data collection, equilibrium can be tested in several ways (1, 10, 11). Developmental equilibrium can be tested in terms of consistent cross-lagged relationships among constructs across different time-lags (e.g., first-order X_k1_ → M_j2_ = X_k2_ → M_j3_, or higher-order X_k1_ → M_j3_ = X_k2_ → M_j4_). Thus, Model-6 constrains the magnitude of the cross-lagged coefficients to be equal across different time-lags; for example Science Efficacy_1_ → Persistence Intentions_2_ = Science Efficacy_2_ → Persistence Intentions_3_ = Science Efficacy_3_ → Persistence Intentions_4_ = Science Efficacy_4_ → Persistence Intentions_5_ (S2 Figure, Model-6). If cross-lagged developmental equilibrium does not worsen model fit, the constraints are carried forward to the next more parsimonious model; however, if the assumption of developmental equilibrium is untenable (i.e., worsens model fit), the constraints are not carried forward to the next model.

In addition, developmental equilibrium can be tested in terms of consistent stability relationships within each construct across different time-lags. Thus, Model-7 constrains the magnitude of the stability coefficients to be equal across different time-lags; for example, Science Efficacy_1_ → Science Efficacy_2_ = Science Efficacy_2_ → Science Efficacy_3_; and
Science Efficacy_1_ → Science Efficacy_3_ = Science Efficacy_2_ → Science Efficacy_4_ (S2 Figure, Model-7). As above, the constraints related to stability developmental equilibrium are carried forward if they do not worsen model fit. Finally, contemporaneous equilibrium can be tested in terms of consistent cross-sectional relationships among constructs across different time-lags. Therefore, Model-8 constrains the magnitude of the cross-sectional coefficients to be equal across different time-lags; for example, Research Experiences_2_ → Science Efficacy_2_ =
Research Experiences_3_ → Science Efficacy_3_ (S2 Figure, Model-8). Finally, after identifying the best fitting most parsimonious model we use a bootstrapping approach to estimate percentile confidence intervals around the longitudinal, reciprocal, and contemporaneous mediated effects (12, 13).

**Results**

**Measurement invariance**

**Longitudinal invariance (within groups)**

Our first goal was to test the longitudinal metric-level measurement invariance of each construct (i.e., science self-efficacy, science identity, science community values, and scientific career persistence intentions). Measurement invariance across time was tested for each construct for men and women separately. As shown in S2 Table, all configural models exhibited acceptable model-data fit using the criteria outlined in the main document. Next, CFA models tested for metric invariance by constraining the factor loadings to be equal across time also exhibited acceptable fit and did not worsen model fit relative to the configural models, S2 Table. The only exception concerned the longitudinal metric invariance models of persistence intentions for both men and women. Close inspection of the local misfit indicators (i.e., standardized residual covariances) indicated that at T1 (high school) the factor loading of indicator 2 was higher than while in college (i.e., T2-T5) and the factors loadings for indicators 3 and 4 were lower than while in college. We settled adjusted the model to allow for partial longitudinal measurement invariance by allowing these indicators factor loadings to be freely estimated. The resulting partial longitudinal measurement invariance model did not worsen model fit relative to the configural invariance model. In sum, the measurement models each construct had exhibited metric-level invariance across time for men and women.

**Cross-group invariance (within time)**

Having established longitudinal measurement invariance for each construct separately for men and women, we next examined the cross-group measurement invariance of all constructs at each time point. Our two-step approach involved first estimating a configural CFA model that allowed factor loadings to be freely estimated in each group at a given time-point (e.g., spring of 1^st^ year in college) and second estimated a metric invariance model that constrained the factor loadings to be equal across groups. For example, a model testing for configural invariance was constructed by including all indicators and constructs for T1 (spring of senior year in high school), allowing men’s and women’s factor loadings to vary. Next, metric invariance was modeled by including all indicators and constructs for T1, but holding all factor loadings to be equal between groups. The test of cross-group measurement invariance was conducted by comparing the change in CFI and RMSEA for configural versus metric models using the criteria described in the main document. As shown in S2 Table, all models of metric invariance provided acceptable levels of global fit and did not worsen model fit compared to the configural models. In sum, the factor structure and factor loadings of all constructs were invariant across groups of men and women at each time point.

**Cross-group correlational invariance (within time)**

As a final step, we tested for invariance of the associations between the constructs at each time point. Functionally, we were interested in determining the degree to which gender may moderate the relationships among the latent constructs. Multiple groups CFA were conducted at each time point, constraining the correlations between latent factors to be equal for men and women. As shown in S2 Table, constraining the correlations between constructs to be equal across groups did not worsen model fit. In sum, the associations among constructs was equal for men and women in the sample at all time-points, which indicates that gender did not moderate the relationships among variables. Gender, therefore, is not considered further in the analyses.

**Tests of longitudinal, reciprocal, and contemporaneous equilibrium**

Next, we tested the full-forward longitudinal and contemporaneous mediation model (Model-1); global indices pointed to relatively good fit (S3 Table). We concluded that Model-1 provided adequate fit to the data and proceeded to test a series of planned simplifications of the full forward model.

Model-2 simplified the longitudinal process by removing all high-order stability and cross-lagged paths. As shown in S3 Table, this extremely simplified longitudinal model resulted in relatively poorer fit (i.e., *ΔCFI* = .068 & *ΔRMSEA* = -.041). Model-3, which retained the first- and higher-order stability paths, as well as the first-order cross-lagged paths, but removed the higher-order cross-lagged paths, did not worsen model fit relative to Model-1 (S3 Table). Model-4, which retained first- and higher order cross-lagged paths, as well as first-order stability paths, but removed the higher order stability paths, resulted in relatively worse model fit (S3 Table). Model-5, which simplified the full forward model by retaining the first- and high-order auto-regressive and cross-lagged paths, but removing the contemporaneous mediated paths worsened model fit (S3 Table). Based on the above series of tests, Model-3 presented the only reasonable simplification of paths for the full forward model.

The next three models were simplifications of Model-3, which estimated first- and higher-order stability, first-order cross-lags, and contemporaneous mediation paths. More specifically, Model-6 tested the assumption of developmental equilibrium by constraining the cross-lagged coefficients to be invariant over time, and the results showed that the simplification did not worsen model fit (S3 Table). Model-7 tested a further aspect of developmental equilibrium by adding the constraint that stability coefficients be equal across time (i.e., cross-lag plus stability constraints). The results indicated that Model-7 worsened model fit (S3 Table). Finally, Model-8 tested contemporaneous equilibrium by adding the constraint that contemporaneous coefficients be equal across time (i.e., cross-lag developmental plus contemporaneous constraints). The results indicated that Model-8 did not worsen model fit (S3 Table). Thus, we concluded that Model-8 provided both adequate fit and improved parsimony.

**Longitudinal, reciprocal, and contemporaneous structural coefficients**

As a final set we examined the structural coefficients estimated in Model 8. Standardized structural coefficients are shown in S4-S7 Tables.

**References**

1. MacKinnon DP. Longitudinal mediation models. Introduction to statistical mediation analysis. New York, NY: Taylor & Francis Group; 2008. p. 193-236.

2. Maxwell SE, Cole DA. Bias in cross-sectional analyses of longitudinal mediation. Psychological methods. 2007;12(1):23-44.

3. Maxwell SE, Cole DA, Mitchell MA. Bias in cross-sectional analyses of longitudinal mediation: Partial and complete mediation under an autoregressive model. Multivariate Behavioral Research. 2011;46(5):816-41.

4. Marsh HW, Byrne BM, Yeung AS. Causal ordering of academic self-concept and achievement: Reanalysis of a pioneering study and ... Educational Psychologist. 1999;34(3):155-67.

5. Arens AK, Marsh HW, Pekrun R, Lichtenfeld S, Murayama K, vom Hofe R. Math self-concept, grades, and achievement test scores: Long-term reciprocal effects across five waves and three achievement tracks. Journal of Educational Psychology. 2017;109(5):621-34.

6. Guay F, Marsh HW, Boivin M. Academic self-concept and academic achievement: Developmental perspectives on their causal ordering. Journal of Educational Psychology. 2003;95(1):124-36.

7. Marsh HW, Craven RG. Reciprocal effects of self-concept and performance from a multidimensional perspective: Beyond seductive pleasure and unidimensional perspectives. Perspectives on Psychological Science. 2006;1(2):133-63.

8. Dwyer JH. Statistical models for the social and behavioral sciences. New York, NY: Oxford University Press; 1983.

9. Kenny DA. Cross-lagged panel correlation: A test for spuriousness. Psychological Bulletin. 1975;82(6):887-903.

10. Marsh HW, Craven RG, Parker PD, Parada RH, Guo J, Dicke T, et al. Temporal ordering effects of adolescent depression, relational aggression, and victimization over six waves: Fully latent reciprocal effects models. Developmental psychology. 2016;52(12):1994-2009.

11. Marshall SL, Parker PD, Ciarrochi J, Heaven PCL. Is self-esteem a cause or consequence of social support? A 4-year longitudinal study. Child Development. 2014;85(3):1275-91.

12. Williams J, MacKinnon DP. Resampling and distribution of the product methods for testing indirect effects in complex models. Structural Equation Modeling: A Multidisciplinary Journal. 2008;15(1):23-51.

13. MacKinnon DP, Lockwood CM, Williams J. Confidence limits for the indirect effect: Distribution of the product and resampling methods. Multivariate Behavioral Research. 2004;39(1):99-128.
