# Supplementary material for: Testing models of reciprocal relations between social influence and integration in STEM across the college years
Source: PLoS One. 2020 Sep 16;15(9):e0238250. doi: 10.1371/journal.pone.0238250 (PMC7494109; doi:10.1371/journal.pone.0238250)
Supplement: Attachment — Submitted filename: PONE-D-20-08469 Review May 2020.docx [file pone.0238250.s012.docx]

Review of “*Testing Models of Reciprocal Relations between Social Influence and Integration in*

*STEM across the College Years*”

This paper presents a theoretically and methodologically sophisticated evaluation of the Tripartite Integration Model of Social Influences (TIMSI) on aspects of STEM participation and persistence for highly represented student demographics in STEM. As outlined by the researchers, the specific analysis presented here aimed to extend previous investigations of TIMSI theory in STEM education by looking at Highly represented rather than Under represented student demographics, by looking at the year before entering college rather than later years in college, and by taking a longitudinal consideration of mentor influences. The study collected five years of self-reported survey data with a final complete (all five years) of survey response rate of 13.7%. This is an overall low response rate but predictable considering that participants were followed for five consecutive years and one would expect a low response rate. Theoretical components of TIMSI were operationalized using a range of psychometrically validated measurement constructs many used in previous studies coming from a psycho-social perspective.

My main concern with this paper is a degree of overstatement of the findings in the last sections of this paper. I would pare back to a certain extent what can or cannot be said in relation to this study and the specific models that is presents. Looking at Figure 3 which model the central SEM relationships found, there are significant pathway coefficients for all variables modelled. However, the pathway coefficients for some constructs are small. In particular, the role of Mentor Network Diversity has three pathways to Science Efficacy (.07), Science Identity (.06) and Science Values (.06). While guidelines of levels of valuable predictive measures are dependent to a certain extent on the theoretical hypothesis that they are predicting, these seem small exactly in relation to the theory presented here. Looking at the model, Faculty Mentor Support influences all three of the psycho-social values of Science Efficacy, Science Identity and Science Values and Research Experiences Influences Science Identity. Furthermore, Science Efficacy on the same criteria has a small predictive relationship with Scientific Career Intentions (.05). The second half of the model does reflect previous research in this area with Science Identity and Science Values have predictive value in terms of Scientific Career Intentions. By looking at the variables with higher predictive value suggests a different and reduced model.

Faculty Mentor Support

Science

Identity

Scientific Career Intentions

Science Values

Research Experiences

In this model, Mentor Network Diversity and Science Efficacy do not have much predictive value based on the empirical results and were accordingly removed in this interpretation. Furthermore, the real value of the study conducted here is situated in the first half of the model. It was already well known from a range of studies that Science Identity and Science Values predict Scientific Career Intentions. It is also known that Research Experiences have a predictive relationship with Science identity and Science Values. The contribution here is the role of Faculty Mentor support over time. Importantly, this variable as opposed to the Faculty Mentor Support variable has, what I consider a measure of the depth of the mentoring relationship. In other words, it increases in accordance with the degree of support actually provided by the faculty mentor. However, as specified in the paper, this influence is limited to contemporaneous effects. In other words, “the impact disappeared when the influence agent was removed” (manuscript p.32. l. 621). As such, we seem to be talking about a model in which social influence plays a role but only in the sense of that year and not as a lasting effect. While no doubt, the researchers here have a far better understanding and experience of their data than presented here in this short interpretation of the presented SEM model, this would suggest a different explanation of the theory that is presented than the one we have at them moment which seems to me to be too influenced by its own theoretical roots and less by the strength of the modelled relationships. Specifically, if Social Influence is limited to a direct yearly influence and the Mentor Network Diversity is not really very predictive, I wonder what this says about the underlying TIMSI theory. This is where I think the final section really overstates what can be said based on this presented model.

The second aspect of the study does present some novel and thought-provoking data. The analysis of longitudinal reciprocal relations present a reversal in current positioning of these variables. In every other study, I have ever looked at using these measurement constructs, Research experiences lead to Science Identity and Scientific Career Intentions. I have no reason to doubt this current analysis and in fact this raises very interesting questions about the way in which Research Experiences are entered into and maintained. The model would suggest that initial aims early in a career direct the sense of being a scientist and the likelihood of entering or finding research experiences. This has some practical ramifications on the idea of student choice within university settings at least within my own work in this area. In general, I very much like the idea presented in this paper on reciprocal relationships and the research does present good evidence on this point.

As clearly stated by the researchers, the analysis presented in the paper is limited to highly represented student populations and as such the situation with other groups might be different in terms of the TIMSI theory. When you reconsider the theoretical explanation provided in relation to the SEM model, I would consider what this says about Highly represented populations in addition to the broader theoretical issues of TIMSI.

In ending I did want to express my admiration for the quality of this study. The theoretical orientation is appropriate and well developed, the operationalization and statistical analysis are excellent and well executed and the paper had me thinking and reflecting for quite a while on what the results actually mean. I recommend moving this paper forward. But I would like the researchers to delve a bit deeper into the theory that emerges from the models presented here.
