# Supplementary material for: Testing models of reciprocal relations between social influence and integration in STEM across the college years
Source: PLoS One. 2020 Sep 16;15(9):e0238250. doi: 10.1371/journal.pone.0238250 (PMC7494109; doi:10.1371/journal.pone.0238250)
Supplement: Attachment — Submitted filename: Response to Reviewers.docx [file pone.0238250.s013.docx]

Dear Dr. Sudzina,

Thank you for the opportunity to revise and resubmit the manuscript entitled, “Testing Models of Reciprocal Relations between Social Influence and Integration in STEM across the College Years” (PONE-D-20-08469). We agree with the reviewer’s generally positive assessments of the potential contributions of this work and we deeply appreciate the constructive feedback on how to improve the manuscript. We have carefully considered the feedback and revised to fully address and improve the narrative and potential contribution of this work. We provide a point-by-point discussion of each comment made by reviewers below (Author responses are shown in bold and italicized text).

Editor:

1. Please ensure that your manuscript meets PLOS ONE's style requirements, including those for file naming. The PLOS ONE style templates can be found at

https://journals.plos.org/plosone/s/file?id=wjVg/PLOSOne_formatting_sample_main_body.pdf and

https://journals.plos.org/plosone/s/file?id=ba62/PLOSOne_formatting_sample_title_authors_affiliations.pdf

***AU: We have reviewed the formatting templates and the manuscript now conforms to the requirements.***

Reviewer #1:

This paper presents a theoretically and methodologically sophisticated evaluation of the Tripartite Integration Model of Social Influences (TIMSI) on aspects of STEM participation and persistence for highly represented student demographics in STEM. As outlined by the researchers, the specific analysis presented here aimed to extend previous investigations of TIMSI theory in STEM education by looking at Highly represented rather than Under represented student demographics, by looking at the year before entering college rather than later years in college, and by taking a longitudinal consideration of mentor influences. The study collected five years of self-reported survey data with a final complete (all five years) of survey response rate of 13.7%. This is an overall low response rate but predictable considering that participants were followed for five consecutive years and one would expect a low response rate. Theoretical components of TIMSI were operationalized using a range of psychometrically validated measurement constructs many used in previous studies coming from a psycho-social perspective.

***AU: We appreciate your concise summary of our study. Of note, we see that multiple reviewers noted a relatively low response rate and we wish to correct that impression by providing more details on the response rates both per-wave of data collection and in terms of the percentage that responded to one or more surveys. Therefore, we now present the wave-by-wave response rate in the “Statistical assumption checking” subsection of the Methods section. Specifically, we now note that the response rate per-survey among the STEM students in our study ranged between 69% and 82% over time. We also discuss the percentage of students responding to one-or-more surveys, as is typical in longitudinal panel studies.***

My main concern with this paper is a degree of overstatement of the findings in the last sections of this paper. I would pare back to a certain extent what can or cannot be said in relation to this study and the specific models that is presents. Looking at Figure 3 which model the central SEM relationships found, there are significant pathway coefficients for all variables modelled. However, the pathway coefficients for some constructs are small. In particular, the role of Mentor Network Diversity has three pathways to Science Efficacy (.07), Science Identity (.06) and Science Values (.06). While guidelines of levels of valuable predictive measures are dependent to a certain extent on the theoretical hypothesis that they are predicting, these seem small exactly in relation to the theory presented here. Looking at the model, Faculty Mentor Support influences all three of the psycho-social values of Science Efficacy, Science Identity and Science Values and Research Experiences Influences Science Identity. Furthermore, Science Efficacy on the same criteria has a small predictive relationship with Scientific Career Intentions (.05). The second half of the model does reflect previous research in this area with Science Identity and Science Values have predictive value in terms of Scientific Career Intentions. By looking at the variables with higher predictive value suggests a different and reduced model.

Faculty Mentor Support

Science

Identity

Scientific Career Intentions

Science Values

Research Experiences

In this model, Mentor Network Diversity and Science Efficacy do not have much predictive value based on the empirical results and were accordingly removed in this interpretation. Furthermore, the real value of the study conducted here is situated in the first half of the model. It was already well known from a range of studies that Science Identity and Science Values predict Scientific Career Intentions. It is also known that Research Experiences have a predictive relationship with Science identity and Science Values. The contribution here is the role of Faculty Mentor support over time. Importantly, this variable as opposed to the Faculty Mentor Support variable has, what I consider a measure of the depth of the mentoring relationship. In other words, it increases in accordance with the degree of support actually provided by the faculty mentor. However, as specified in the paper, this influence is limited to contemporaneous effects. In other words, “the impact disappeared when the influence agent was removed” (manuscript p.32. l. 621). As such, we seem to be talking about a model in which social influence plays a role but only in the sense of that year and not as a lasting effect. While no doubt, the researchers here have a far better understanding and experience of their data than presented here in this short interpretation of the presented SEM model, this would suggest a different explanation of the theory that is presented than the one we have at them moment which seems to me to be too influenced by its own theoretical roots and less by the strength of the modelled relationships. Specifically, if Social Influence is limited to a direct yearly influence and the Mentor Network Diversity is not really very predictive, I wonder what this says about the underlying TIMSI theory. This is where I think the final section really overstates what can be said based on this presented model.

***AU: Thank you for carefully pointing out (a) where we failed to closely align our findings with the magnitude of the effects, which resulted in over-interpretation of some small effects for network and efficacy, and (b) for presenting a thoughtful alternative model based on the data. Concerning over-interpretation, we agree that we need to temper our findings and discuss them in light of their relative effect sizes. To that end, we have now made major revisions to the Discussion section to align our interpretations with relative strength of effects and also noting how mentor network diversity and efficacy play a very small role in the role influence process over the college years (see “Social influence leads to student integration” subsection of the Discussion section). We also now expanded and contextualize the mentor network diversity findings in light of other work on mentor network characteristics. Specifically, we propose that alternative aspects of network characteristics (e.g., triadic structures) may be more strongly associated with integration and that future research should examine other network structures as more impactful instances of social influence. Furthermore, you made an important point about emphasizing the short rather than long-term effects of the social influence process leading to integration. We have now expanded our discussion of the social influence process in light of Kelman’s work and theorizing on short-term social influence (see “Social influence leads to student integration” subsection).***

***To your second point, we have now expanded our discussion of how the TIMSI model plays out over the college years. Specifically, we interpret these findings as reflecting developmental process where the mechanisms for social influence change over time. Specifically, we discuss and point to research indicating that self-efficacy is a prominent mechanism early in the development of career interests; identity and to a lesser extent values are prominent mechanisms in the college years; and values are a prominent mechanism in graduate school and among postdocs. This developmental interpretations is consistent with the present data, as well as other works, and provides more nuanced hypotheses to be tested in future work testing TIMSI-based hypotheses.***

The second aspect of the study does present some novel and thought-provoking data. The analysis of longitudinal reciprocal relations present a reversal in current positioning of these variables. In every other study, I have ever looked at using these measurement constructs, Research experiences lead to Science Identity and Scientific Career Intentions. I have no reason to doubt this current analysis and in fact this raises very interesting questions about the way in which Research Experiences are entered into and maintained. The model would suggest that initial aims early in a career direct the sense of being a scientist and the likelihood of entering or finding research experiences. This has some practical ramifications on the idea of student choice within university settings at least within my own work in this area. In general, I very much like the idea presented in this paper on reciprocal relationships and the research does present good evidence on this point.

***AU: Thank you, we agree.***

As clearly stated by the researchers, the analysis presented in the paper is limited to highly represented student populations and as such the situation with other groups might be different in terms of the TIMSI theory. When you reconsider the theoretical explanation provided in relation to the SEM model, I would consider what this says about Highly represented populations in addition to the broader theoretical issues of TIMSI.

***AU: We have now revised our discussion of TIMSI theory to more fully address the comments above, about the dominant emergent patterns of social influence, as well as contextualizing this work in light of the focus on students from HO groups in STEM. We more thoroughly provide this contextualization in the 1^st^ paragraph of the Discussion section, in the 2^nd^ paragraph of the “Longitudinal findings depart from earlier longitudinal research” subsection of the Discussion, and in the “Limitations and implications for future research” subsection of the Discussion.***

In ending I did want to express my admiration for the quality of this study. The theoretical orientation is appropriate and well developed, the operationalization and statistical analysis are excellent and well executed and the paper had me thinking and reflecting for quite a while on what the results actually mean. I recommend moving this paper forward. But I would like the researchers to delve a bit deeper into the theory that emerges from the models presented here.

***AU: Thank you. We have taken your advice seriously and have more carefully considered and revised our interpretation of the results, as well as the TIMSI framework in light of these results throughout the Discussion section.***

Reviewer #2:

This paper presents the results of a longitudinal study spanning the entirety of undergraduates’ careers and investigating the Tripartite Integration Model of Social Influences (e.g., how Social Influence Agents affect Social Influence Processes which may intern influence Social Integration and other relationships among these factors). Their results indicate that more diverse mentor networks and high quality faculty mentorship support and research engagement support STEM integration through (mediated by) their influence on science identity and science community values. In addition the authors find that high levels of initial integration predict pursuit of additional research experiences via the effect on science identity.

I find this paper to adhere to all standards and metrics for publication in PLOS one. Experiments and analyses are performed to a high technical standard, conclusions are appropriate and claims are aligned with results, and the study clearly identifies a gap in the literature. Furthermore, the writing is clear, concise, and while it is technically complex at times, I feel it would be accessible to a reader who is not overly familiar with SEM and the other techniques used.

***AU: Thank you, we agree.***

Responses to the following minor comments would improve the paper:

Line 127. You have not yet discussed the process of reciprocal influence in the manuscript, clarify for what and whom the influence could be reciprocal.

***AU: Thank you for pointing out this omission. We now more fully discuss what and for whom the influence could be reciprocal (see end of the “Social influence and student integration” subsection of the Introduction). Specifically, reciprocal influence in the TIMSI framework operates when persons desiring to integration into a social system self-socialize into that system by seeking out experiences with agents of the social system.***

Lines 214-218 It may be worth mentioning in this paragraph why you did not use the data set you drew on for this study, which includes HU students, to also analyze HU students and compare their outcomes to HO students. Was this a sample size issue?

***AU: This is an important point. We now more fully elaborate on our rationale for conducting the study focused on HO students. This was both an issue of (a) theoretical extension of prior research to subgroup where TIMSI predications had not been tested and (b) an issue of sample size were it was impossible to (i) test measurement invariance across demographic groups and (ii) directly test for similarities/differences across subgroups via multiple-groups SEM. Any attempt to compare groups where measurement invariance has not been established risks the threat of confounding real group differences with systematic measurement error (i.e., differential item/test function or measurement bias). We were loath to commit this well-known error. Therefore, we now acknowledge both points now in the "Current Study" subsection of the Introduction. And we continue to point to this as a limitation of the present research (Discussion section), as well as, point to the need for further study in directly comparing TIMSI predictions across identifiable subgroups, rather than across studies as was done in the present case.***

Line 276 You explain that 22% of your HO sample were first-generation college students. In many studies, these students are reported as underserved or underrepresented. Could you comment on why these students are included in the HO sample?

***AU: This is an interesting point. We primary operationalized historical over and underrepresentation concern gender and race/ethnicity, as there is robust research showing the persistent representation gap in some STEM disciplines (e.g., NSF reports on Women, Minorities, and Persons with Disabilities in Science and Engineering). The gap for first-generation students in STEM less well studied. However, recent nationally representative research on STEM degree attainment comparing first- and multi-generational students has shown that the first-generation effect disappears when pre-college experiences are accounted for (Bettencourt et al., 2020). This study is unique, in that we have captured all variables in the model as pre-college experiences and perceptions (i.e., our first data collection period was in the spring/summer prior to college). Based our research design, we argue that we have accounted for precollege experiences influence on the TIMSI process. Therefore, first- versus multi-generational group differences are very unlikely to have influenced our findings. That being said, we now also acknowledge the need for additional study on the degree to which TIMSI derived hypotheses hold over first- versus multi-generational students in the Limitations subsection of the Discussion.***

***Bettencourt, G. M., Manly, C. A., Kimball, E., & Wells, R. S. (2020). STEM Degree Completion and First-Generation College Students: A Cumulative Disadvantage Approach to the Outcomes Gap. The Review of Higher Education, 43(3), 753-779.***

Table 1. It may not be immediately clear to readers who do not frequently do statistics what N, M, and SD mean. Perhaps add these to the notes at the end of your table. In addition, please briefly explain how to interpret the numbers for N as these are not intuitive.

***AU: Per your suggestion, we now explain the meaning of each of the statistical notation terms within the Table 1 notes and we provide a brief explanation of how to interpret the N values within the table in the context of demographic examples (see “Participants” subsection of the Methods section)***

Lines 357-369. Please provide an explanation for why the pre-college survey responses and college survey responses differ substantially. It is clear why ‘university faculty’ are on one survey and ‘teachers’ are on the other, but it is not clear why family members and others were removed from the second scale.

***AU: We now briefly explain the slight differences in the indicator of mentor network diversity in the Measures subsection of the Methods. Specifically, our pre-college network diversity index was intended to capture mentors across a full range of contexts prior to college. However, the college survey focused on potential mentors within the college context, as these mentors are more proximal to the context and focus of the study.***

Line 504 and Table 2. The p value cutoff you use in your table is 0.023 and it appears that research experiences did not make this cutoff for predicting scientific career persistence intentions as indicated by the absence of an * in the table (unless I am somehow reading the table incorrectly). Do you still want to discuss this result? ...or perhaps you just need to add the “*” in the table.

***AU: Thank you, we have corrected this error throughout the results and discussion sections.***

Table 2.

-Should the first listing of “Research Experiences” under T5 4th Year of College Outcomes actually be “Persistence Intentions”?

***AU: Thank you for pointing out some ambiguity in Table 2. We have updated this Table to be consistent with PLOS ONE formatting and to correct the ambiguity.***

- It gets a little challenging to remember which components are “Social Influence Agents” and “Social Influence Processes.”. Could you somehow designate “agents” and “processes” in the table to remind readers of these components?

***AU: Good point. We have done so by adding table headers that clearly identify the outcome, agents, and processes.***

Lines 494-535

-It would be nice if you created a supplementary structural equation model figure that would present a schematic representation of the data in Table 2 and referenced it somewhere in your descriptive text. For readers who are helped by diagrams and schematics of data, this would elucidate relationships between variables. This figure would be a bit complex and large (hence the recommendation to include it in the supplement), but I feel it would help readers to interpret the results and view the data from a temporal perspective.

***AU: Thank you for this recommendation. We have created a new supplemental figure (S3 Fig.) and referenced it in text (see “Social influence operates contemporaneously and reciprocal influence operates longitudinally” subsection of the Results).***

-It is a little counter intuitive having the contemporaneous coefficients discussed first when they are in the second part of the table. Could you cue the reader to look in the lower section of the table for these results in the text so that it is easier to cross-reference?

***AU: Thank you for this feedback. We have done so in the second sentence of the “Social influence operates contemporaneously and reciprocal influence operates longitudinally” subsection of the Results.***

Line 615. Consider also referencing the earlier call: <https://www.lifescied.org/doi/full/10.1187/cbe.11-03-0028>

***AU: Thank you for directing our attention to this important earlier call. We have done so.***

Lines 617-624 - Did you explore the possibility that influence operates more rapidly for HO students than for HU students? Did the prior literature that you refer to here explore HO or HU students? This may be worth commenting on.

***AU: This is an interesting question and would like to see it addressed. Unfortunately, it was not possible to adequately test this question in the present study. Furthermore, given the state of the extant literature, no other studies have been positioned to think of or test this question. That is, prior studies have not distinguished between contemporaneous and longitudinal influence, nor have they compared HO and HU groups across these differential pathways of influence. We now comment on this interesting research question in the Limitations and Future Directions subsection of the Discussion.***

Lines 626-644 - Do you have any hypotheses as to why values, specifically, are a mediator for HO students but not for HU students? Can you find or point to any literature that explores the differences between values and identity and discusses how these two social processes may be acting in order to explain your results?

***AU: Given the feedback from Review #3, we have tempered some of our inferences on this issue. Specifically, we have found different patterns from prior research; however, these different patterns across groups are confounded with different methodological approaches. Therefore, although we still discuss different patterns found here compared to those found among HU groups, we contextualize them in light of those methodological differences and make note that discovery of true subgroups differences need to do so while accounting for methodology. Given the limited nature of the extant literature, we are unable to point to studies that explore why the pattern of processes may be operating differently. This is an area where we think future study benefit and clarify these findings. We discuss these issues in the 2^nd^ paragraph of the “Longitudinal findings depart from earlier longitudinal research” subsection of the Discussion.***

Grammatical/Wording Suggestions:

Line 179 - It may make more sense to say ‘processes’ instead of ‘process’

Line 226 - Change ‘design’ to ‘designed’

Line 499 - Please add the word “and” between “self-efficacy” and “science identity”

***AU: Thank you for catching these typos. They have been corrected.***

Reviewer #3:

This study examined longitudinal links among mentoring, psychological processes, and science career intentions among historically overrepresented university students. The paper has many strengths, and in particular I appreciated the rigorous mediation and model selection methods along with the ambitious goals to comprehensively test these processes. However, I have a moderate to serious concerns about the rationale for the study, the methods, and the results as outlined in detail below:

1. I wondered why the focus was on historically overrepresented groups. Prior studies having largely examined HU groups (lines 170-180) is not really an adequate rationale for the exclusion of HU students from the sample. Further, if an aim of the study is to identify subgroup differences/similarities (line 217-218), it makes more sense to include the subgroups in the current study. Currently, the study does not allow for direct comparisons, so differences between this study and prior studies could be attributed to the many differences in the data and study design that were earlier identified as unique strengths of this paper.

***AU: Thank you for your feedback. We partially agree. Specifically, we would disagree in that focusing on understudied groups to test TIMSI hypotheses (or to test hypotheses from other theoretical models) is in and of itself an important process and a well-grounded rationale. However, we agree that we need to more fully elaborate on our rationale for conducting the study focused on HO students. This was both an issue of (a) theoretical extension of prior research to subgroup where TIMSI predications had not been tested and (b) an issue of sample size were it was impossible to (i) test measurement invariance across demographic groups and (ii) directly test for similarities/differences across subgroups via multiple-groups SEM. Any attempt to compare groups where measurement invariance has not been established risks the threat of confounding real group differences with systematic measurement error (i.e., differential item/test function). Therefore, we now acknowledge both points now in the "Current Study" subsection of the Introduction. And we continue to point to this as a limitation of the present research (Discussion section), as well as, point to the need for further study in directly comparing TIMSI predictions across identifiable subgroups, rather than across studies as was done in the present case.***

2. The rationale for the need and purpose of this study might be greatly strengthened by mentioning a specific theory or theories and their tenets rather than relying broadly on the propositions and potential extensions of “social psychology theories” (e.g., lines 69, 82, 666). Social cognitive models, for example, would be a more specific framework to draw upon, or expectancy-value theory in particular seems very relevant to this study.

***AU: Thank you for this point. We have elaborated on our discussion of social psychology theories by pointing to one widely used theoretical model, that is, Social Cognitive Career Theory (SCCT). SCCT has been widely used to understand and predict STEM persistence behaviors and enriches our discussion of TIMSI as an alternative theoretical approach (see the “The rise of mentorship as an antidote to attrition” subsection of the Introduction).***

3. I also have concerns about the measures. First, the reliabilities are a bit low for scientific career intentions. Second, why were measurement analyses presented for some but not all constructs (e.g., why was the faculty mentoring support measure not included in the CFAs or tests of measurement invariance)? Third, what was the rationale for the use of composite (mean) scores rather than latent constructs or factor scores? Lastly, all measures are self-report, introducing the strong possibility of over-inflated relations among variables due to common method bias.

***AU: Regarding the reliability of the intentions variable, we too would always like to see the highest possible reliability coefficients. However, in light of all of the other information provided in this manuscript, we argue that the observed level of reliability is acceptable. As discussed by Schmitt (1996), reliability is only one of many factors that must be used to determine the quality of measurement. In the present case, we have provided strong evidence through CFAs that (a) intentions are a unidimensional construct (which is a prerequisite of accurate interpretations of reliability coefficients… as noted by Schmitt), (b) that the property of unidimensionality holds over time, (c) that the construct is measured equivalently for both men and women, (d) and that the construct of intentions is distinguishable or has discriminant validity from the other constructs under study. When there is robust evidence for the adequacy of measurement, such as those presented in this paper, even very low reliability (e.g., .50) does not threaten validity coefficients (see Schmitt, 1996).***

***Thank you for bringing the issue of measurement of mentorship to light. We inadvertently omitted a discussion of this issue in the prior manuscript and have now addressed this important issue. We have now revised our discussion on the measurement properties of the global measure of mentorship practices, a well-established measure of mentorship support. We now discuss in detail in that we were unable to conduct CFA and measurement invariance tests on this scale due to the ratio of parameter estimates to the sample size of students with a faculty mentor (see “Longitudinal and cross-group measurement invariance CFA models” subsection of the Methods section). Specifically, the CFA models involving the of global measure of faculty mentorship practices had far more parameter estimates than persons on which to estimate the models, which results in non-converging / non-estimable models. Therefore, we proceed by reporting on the reliability coefficients for this well-established measure of mentorship support.***

***Your point about composite scores is interesting and well taken. Ideally, we would have liked to conduct this study in a fully latent framework for all of the latent constructs. However, given the number of constructs, number of items per construct, and number of time points, a fully latent model would have resulted in an exceptionally large number of parameter estimates- far larger than our robust sample size would make possible to estimate. Your suggestion of using factor scores, as an alternative to composite scores, is one approach; however, this approach is methodologically controversial and potentially problematic due to the issue of factor score indeterminacy (Grice, 2001). Furthermore, it would be a major deviation from the prior literature with these scales to transition from a composite or mean scale score to that of a factor score approach. Given that one of our goals was to extend the literature, we argue that there is a more compelling argument to keeping similar scoring procedures as used in prior studies (which were based on mean scale scoring), rather than based on factor-based scale scores. Therefore, based on the realities of our sample size and the issues with factor scale scoring, we have decided to retain our current approach to the measurement/scoring of scales in this study.***

***Lastly, we agree that more diverse operationalization’s of our measures would improve this study. Specifically, we have expanded our discussion on the limitations and future directions for research. We now extend our discussion to emphasize the need for future studies to incorporate additional non-self-reported instruments (see the Limitations and future directions subsection of the Discussion section).***

***Schmitt, N. (1996). Uses and abuses of coefficient alpha. Psychological Assessment, 8(4), 350-353. doi:10.1037/1040-3590.8.4.350***

***Grice, J. W. (2001). Computing and evaluating factor scores. Psychological Methods, 6(4), 430-450. doi:10.1037/1082-989X.6.4.430***

4. Missing data presents another major concern. There is a great deal of missing data, on some variables greater than 80%. Importantly, there was no evidence presented for the MAR assumption (line 416) that must be met for FIML estimation to produce unbiased estimates. Reasons for missing data are clearly non-random as, for example, students did not complete the mentoring practices measure if they did not have a university faculty mentor. It does not seem appropriate to estimate relations of mentoring quality with the other variables for students who did not experience university faculty mentoring at all. Further, the available evidence (e.g., demographic percentages) does not support the claim that “participants largely mirror the demographics of the university” (line 259-260), particularly within this analytic sample (e.g., 58% female in the study vs. 51% female at the university; entire racial/ethnic groups were excluded).

***AU: Thank you for this note and the point is well taken. Both you and reviewer #1 noted missing data, which is a common issue in longitudinal studies. We have now added to our discussion missing data and clarified the issue of missingness. Specifically, we show that we had acceptably high response rates wave over wave throughout the study (average response rate was ~77%). We have also now expanded our discussion to make clear that violation of the MAR assumption is very unlikely under longitudinal panel designs, such as the one used here. We expanded our discussion of the MAR assumption, as well as why longitudinal panel designs are unlikely to violate this assumption (see the first paragraph of the Statistical assumption checking subsection of the Methods section). Specifically, MAR allows missing data when the missing values are conditioned on observed variables in the analysis. In longitudinal panel designs, like this one, missing data at future waves can be conditioned on the values of variables collected at prior waves. This approach makes it highly unlikely that the MAR assumption is violated.***

***Your point about missing data in the faculty mentorship practices measure is well taken. However, we argue that this is not actually problematic in this study because the mechanism of missing data (i.e., the factor that causes data to be missing or not) is captured and included in our present models and functionally serves as an auxiliary variable (Enders, 2008, 2010). That is, our index of mentorship network diversity includes whether or not the participants had a faculty mentor in their network. By including this variable in our model, we are confident that the estimates are adjusted for the covariate of missing data (i.e., the auxiliary variable) and that missing data bias is not problem in these data.***

***Finally, thank you for pointing out the confusion caused by our statement on demographics. We have further contextualized our statement to note that prior research from the larger longitudinal study showed a high degree of proportional representativeness between the university class of 2019 and the study sample (see Participants subsection of the Methods section). This is just meant to point readers to the overall context of the study. However, we also now note that our present study does not involve the full larger study sample, but focuses instead on the subsample of concern for this study, i.e., HO students in STEM majors. We hope that this clarifies our description of the sample.***

***Enders, C. K. (2008). A note on the use of missing auxiliary variables in full information maximum likelihood-based structural equation models. Structural Equation Modeling- A Multidisciplinary Journal, 15(3), 434-448. doi:10.1080/10705510802154307***

***Enders, C. K. (2010). Applied missing data analysis. New York, NY: Guilford Press.***

5. The modelling approach is in need of more detailed explanation and rationale in the text. For example, it was unclear whether the many variables were modeled in separate models or together in one model, or whether this was the most appropriate approach given the potential for multicollinearity, higher-order factor structures, etc.

***AU: We appreciate this comment. We have attempted to strike the right balance between providing sufficient information in the main narrative and more copious information in the supplemental materials. In responding to this comment, we have expanded our explanation of our longitudinal modeling approach in both the Current study subsection of the Introduction and in the Results section. Specifically, we now provide details in the Current study subsection on the best practices approach to longitudinal and reciprocal mediation modeling (e.g., MacKinnon, 2008; Marsh et al., 1999). We still refer readers to the supplemental materials for more copious information on the background of this modeling approach. Furthermore, we walk readers through an interpretation of our final model (i.e., Model-8) in the Results. We hope that this makes clear that all variables were put together into a single model. Furthermore, we would argue that multicollinearity and higher order factor structures are not a problem for this study, in that we have provided robust evidence of discriminant validity via (a) conceptual/theoretical distinctions across constructs, (b) the CFA modeling results, and (c) the correlation matrix wherein scale scores showed small-to-moderate correlations across constructs.***

***MacKinnon DP. Longitudinal mediation models. Introduction to statistical mediation analysis. New York, NY: Taylor & Francis Group; 2008. p. 193-236.***

***Marsh HW, Byrne BM, Yeung AS. Causal ordering of academic self-concept and achievement: Reanalysis of a pioneering study and ... Educational Psychologist. 1999;34(3):155-67.***

6. The results are very hard to understand because there are so many processes under consideration and because of the aforementioned lack of clarity in the modelling approach. I recommend more deliberately explaining each finding in both technical terms as well as plain-language interpretations of the meaning of the findings to help readers more easily digest the results. Due to the lack of clarity, it was difficult to determine how well the results support conclusions such as “social influence operates rapidly” (line 616).

***AU: Thank you for your request for additional clarification. We have added to our interpretations throughout the results section to emphasize both the technical and the plain language interpretations of our findings. More specifically, we now include a more detailed review of the models results in each of the main emphasis areas (contemporaneous, longitudinal, and reciprocal). Furthermore, we include many more examples of explanations of specific paths that include both technical and plain language interpretations. Per Reviewer #2, we have also now included a new figure (Fig S3) to compliment the results shown in Table 2. We appreciate the feedback and believe these changes will make the results much more comprehensible.***

7. What criteria (e.g., statistical test) were used to determine which is the “strongest” predictor (e.g., lines 502, 506, 544)?

***AU: We agree that we should have been explicit about our interpretations of relatively stronger and weaker predictors. We have updated our results section to point out that we are using the relative magnitude of the standardized predictors to guide our interpretations of the relative strength of different predictors. Moreover, consistent with Reviewer #1, we also now include much more detail on the relative magnitude of different standardized coefficients and we carry this into the Discussion section.***

8. The writing is generally strong, however a number of typos indicate the need for a thorough proofreading. For example:

(line 191-192) “STEM majors with a larger and more diverse networks”;

(line 208-209) “we hypothesized that the level faculty support”;

(line 437) “persistence intensions”; (line 505-506).

***AU: Thank you for catching these typos. These and others have now been fixed.***
